# Soyabean response to rhizobium inoculation across sub-Saharan Africa: Patterns of variation and the role of promiscuity

**DOI:** 10.1016/j.agee.2017.08.016

**Published:** 2018-07-01

**Authors:** Joost van Heerwaarden, Frederick Baijukya, Stephen Kyei-Boahen, Samuel Adjei-Nsiah, Peter Ebanyat, Nkeki Kamai, Endalkachew Wolde-meskel, Fred Kanampiu, Bernard Vanlauwe, Ken Giller

**Affiliations:** a Plant Production Systems, Wageningen University, P.O .Box 430,6700. AK, Wageningen, The Netherlands; b International Institute of Tropical Agriculture (IITA), Dar es Salaam, Tanzania; c IITA, Nampula, Mozambique; d IITA Tamale, Ghana; e IITA Kampala, Uganda; f IITA Kano, Nigeria; g International Livestock Research Institute, Addis Ababa, Ethiopia; h IITA, Natural Resource Management Research Area, Nairobi, Kenya

**Keywords:** Bradyrhizobium, Soyabean, Smallholder farmers, Sustainable intensification, Sub saharan Africa, Promiscuous varieties, Response variability

## Abstract

Improving bacterial nitrogen fixation in grain legumes is central to sustainable intensification of agriculture in sub-Saharan Africa. In the case of soyabean, two main approaches have been pursued: first, promiscuous varieties were developed to form effective symbiosis with locally abundant nitrogen fixing bacteria. Second, inoculation with elite bacterial strains is being promoted. Analyses of the success of these approaches in tropical smallholder systems are scarce. It is unclear how current promiscuous and non-promiscuous soyabean varieties perform in inoculated and uninoculated fields, and the extent of variation in inoculation response across regions and environmental conditions remains to be determined.

We present an analysis of on-farm yields and inoculation responses across ten countries in Sub Saharan Africa, including both promiscuous and non-promiscuous varieties. By combining data from a core set of replicated on-farm trials with that from a large number of farmer-managed try-outs, we study the potential for inoculation to increase yields in both variety types and evaluate the magnitude and variability of response.

Average yields were estimated to be 1343 and 1227 kg/ha with and without inoculation respectively. Inoculation response varied widely between trials and locations, with no clear spatial patterns at larger scales and without evidence that this variation could be explained by yield constraints or environmental conditions. On average, specific varieties had similar uninoculated yields, while responding more strongly to inoculation. Side-by side comparisons revealed that stronger responses were observed at sites where promiscuous varieties had superior uninoculated yields, suggesting the availability of compatible, effective bacteria as a yield limiting factor and as a determinant of the magnitude of inoculation response.

## Highlights

• The effect of inoculation was evaluated in 2082 on-farm soyabean trials across Africa.
• Significant but moderate responses were observed.
• Variability was high and largely unexplained by considered environmental factors.
• Promiscuous varieties had similar yields but lower responses than specific types.
• Strong responses coincided with better uninoculated yields of promiscuous varieties.

## 1 Introduction

Increasing production and productivity of grain legumes are widely recognized as important components of sustainable intensification strategies for sub-Saharan Africa (SSA) (Vanlauwe et al., 2014). The ability of legumes to use atmospheric nitrogen fixed by symbiotic rhizobial bacteria, offers the potential for improving yield without nitrogen fertilizer. In soyabean, nitrogen fixation can only occur in the presence of compatible bacterial strains, typically of the genus *Bradyrhizobium*. Soyabean is an Asian crop with a relatively brief history of cultivation in Africa (Mpepereki et al., 2000). Many improved varieties developed for SSA derive from North American stock and were thought to establish symbiosis with only one *Bradyrhizobium* species, *B. japonicum*, that was assumed not to occur natively in African soils (Kueneman et al., 1984). Field tests performed in the 1970s and 80 s (Chowdhury, 1977, Nangju, 1980, Pulver et al., 1982, Pulver et al., 1985, Kueneman et al., 1984) suggested that the absence of compatible *B. japonicum* in African soils may limit nitrogen fixation and productivity of such varieties.

Two main strategies were followed to address this problem (Sanginga et al., 1997). First, public breeding since the late 1970s focused on the development of promiscuous varieties that can fix nitrogen effectively with locally abundant bacteria (Kueneman et al., 1984). Second, use of inoculants, containing elite strains of *B. japonicum*, has been promoted in combination with both specific and promiscuous varieties (Gyogluu et al., 2016, Ronner et al., 2016). Despite a wealth of studies on inoculation responses in soyabean in SSA (Nangju, 1980, Osunde et al., 2003a, Osunde et al., 2003b, Sanginga et al., 1996, Sanginga et al., 1997, Bromfield and Ayanaba, 1980, Okogun and Sanginga, 2003, Okogun et al., 2005, Thuita et al., 2012, Ronner et al., 2016, Gyogluu et al., 2016) there is currently no consensus on the general need for inoculation or on the benefits of planting promiscuous over specific varieties. Although several studies suggest that promiscuous varieties may not benefit much from inoculation (Pulver et al., 1985, Okogun and Sanginga, 2003, Osunde et al., 2003b), others have observed significant inoculation effects (Ronner et al., 2016, Mpepereki et al., 2000, Gyogluu et al., 2016). Furthermore, the assumption that specific varieties yield poorly in the absence of inoculation is not always supported by the evidence (Pulver et al., 1982, Gyogluu et al., 2016).

One complicating factor has been the inherent variability of inoculation responses (Ronner et al., 2016) and the dependence of biological nitrogen fixation on agronomic, environmental and edaphic factors (Graham, 1981, Graham et al., 1994, Hardarson, 1993). Aspects such as source of inoculant, varieties and management practices are likely to be different across countries, while differences in soil and climatic conditions exist across different spatial scales. This variability makes it harder to draw general conclusions on the efficacy of inoculants from local experiments and suggests that recommendations regarding the use of inoculants and promiscuous varieties may need to be tailored to specific contexts (Ronner et al., 2016). Thus, there is a clear need for comparative studies into the effectiveness of these two legume technologies that encompass soyabean growing areas across SSA.

Here we present the results of an analysis of data obtained from a total set of 2082 soyabean trials performed in ten countries (D.R. Congo, Ethiopia, Ghana, Kenya, Malawi, Mozambique, Nigeria, Rwanda, Uganda and Zimbabwe) from 2010 to 2015. The trials consisted mainly of on-farm try-outs with a single replicate per farm in addition to a smaller set of replicated, researcher managed, agronomy trials which were designed to test the effect of inoculation, phosphorus fertiliser, varieties or their combination. This wide array of data allowed us to address a number of basic questions about the response to inoculation across SSA. First, we wish to quantify the overall response to inoculation in soyabean, to measure its variation and to analyse how this variation is partitioned geographically and in relation to climatic and edaphic factors. Second, we compare the relative benefits of using promiscuous varieties in soils with and without inoculation, to establish the extent to which these benefits differ between countries and locations. We thereby hope to contribute to discussions on the suitability of soyabean technologies across SSA.

## 2 Materials and methods

### 2.1 Field trials

**Fig. 1:**
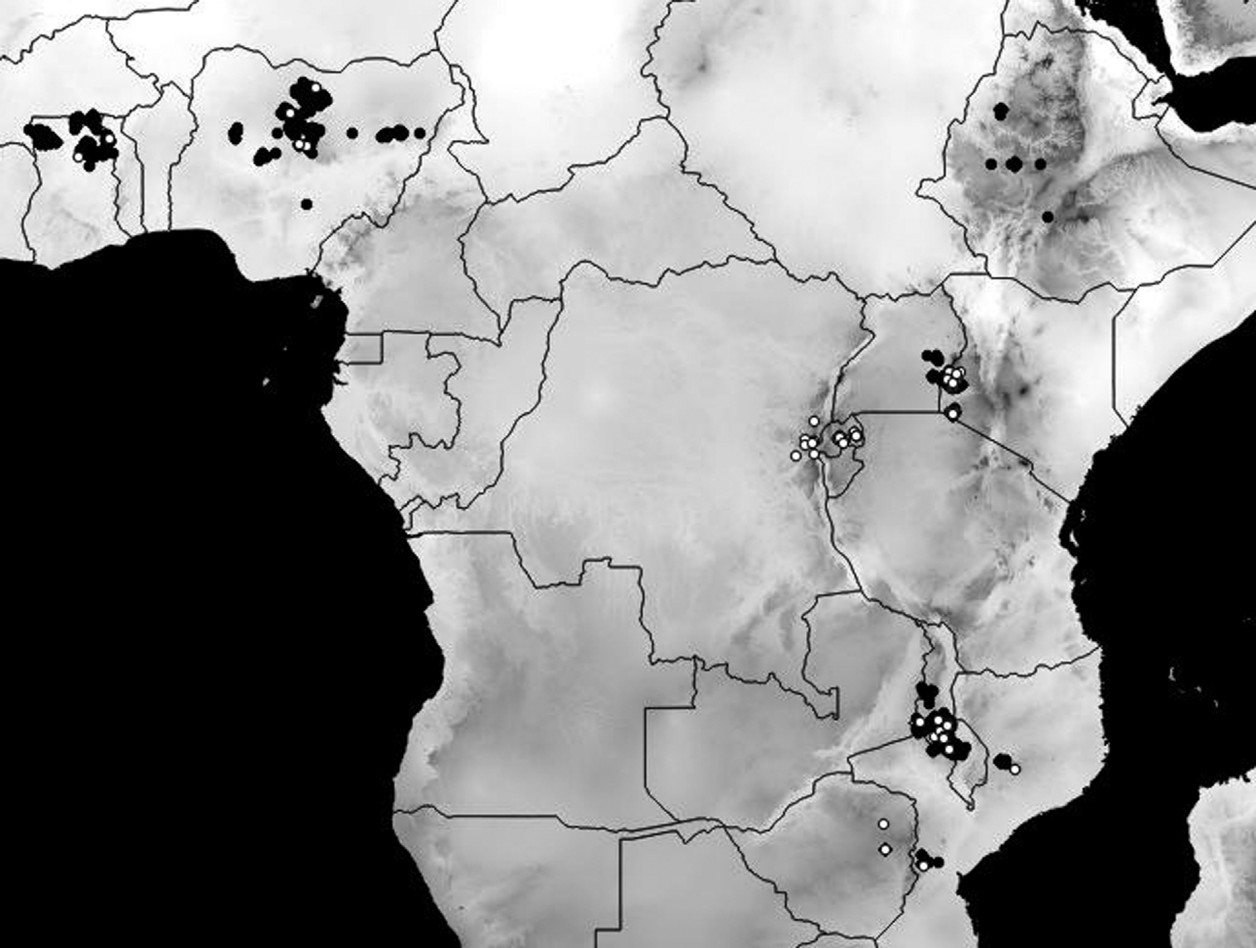
Map of trial locations for which coordinates were available. Black: on-farm try outs, White: replicated agronomy trials.

From 2010 to 2015 a diverse set of on-farm trials was implemented to study soyabean yield and response to inputs across ten countries, spanning three major agro-ecological zones (AEZ) of sub-Saharan Africa (DR Congo, Ethiopia, Ghana, Kenya, Malawi, Mozambique, Nigeria, Rwanda, Uganda, Zimbabwe) (Fig. 1). In West Africa, specific action sites were Northern Ghana (Northern and Upper West Regions), falling within the southern Guinea Savanna, and Northern Nigeria (Kano, Kaduna, Zaria and Borno States), falling within the northern Guinea Savanna. In Central Africa sites were located Rwanda (Eastern and Southern provinces) and Eastern Democratic Republic of Congo (Walungu and Kabale territories), while East Africa included sites in West Kenya (former Western and Nyanza provinces), Uganda (four districts in the north) and Ethiopia (Eleven districts across the country). In Southern-Africa, action sites covered parts of Malawi (Lilongwe, Dedza and Salima), Zimbabwe (Wedza, Chegutu, Murewa, Makoni west and Mudzi districts) and Mozambique (Tete, Zambesia and Manica provinces). The final dataset consisted of 122 on-farm, researcher designed and researcher/farmer co-managed, agronomy trials with a varying number of replicates on-site and 1960 un-replicated on-farm try outs that were researcher designed and farmer managed.

Treatments varied between trials but typically included phosphorus fertiliser, inoculants, promiscuous and specific varieties in different combinations (see Table S1 for a summary of the main trial types). Different rhizobia inoculants were used, depending on the availability in each country: Biofix (MEA Kenya Ltd) and MakBiofixer (Makerere University), containing *Bradyrhizobium* stain USDA 110, Legumefix (Legume Technology, UK), containing strain 532c, Soycap (Soygro Ltd), containing strain WB74, MBI inoclulant (Menagesha Biotech Industry), containing strain TAL379, and an inoculant by Soil Productivity Research Laboratory in Zimbabwe (SPRL), containing MAR1391 (Table S1). A wide range of varieties was used, all of which were categorized as being either promiscuous or specific (non-promiscuous) (Table S2).

Plot sizes varied but were typically around 18 m^2^, with a 12 m^2^ net-plot for agronomy trials and 100 m^2^ for on-farm try-outs. In agronomy trials, dry weight of a subsample of grain was determined to adjust grain yield to 13% moisture. In the case of on-farm try outs, field-dry grain weight was used as an estimate of grain-yield. For a subset of agronomy trials, biomass accumulation was determined at pod formation stage by harvesting the plants from an area of 0.5 m^2^, randomly selected at one corner of each plot. Root nodules were counted in a number of trials, after carefully uprooting and washing.

### 2.2 Statistical analysis

Linear mixed models, as implemented in ASREML-R (Butler et al., 2009), were used to estimate the effects of different variables of interest while controlling for possible sources of error and confounding. Replicates (rep) and main plots (mpf) were taken into consideration as random effects where applicable and different residual errors were estimated for the two types of trials. The latter is important since the two types of trials differed in aspects of their implementation such as harvest area which may affect plot-level variance. Experimental units with absolute residuals larger than four standard deviations were removed from the data, as were trials with average yields above 4000 kg/ha or average responses above 1000 kg/ha. Significance of fixed effects was determined by a Wald-test.

What follows is a description of the different models applied, where the choice of fixed and random factors reflects differences in the effects to be estimated and the design features of the data involved. Square brackets with subscripts indicate terms that differ between different sub-models.

The effect of inoculation (I), phosphorus fertiliser (P), promiscuity, country or country/year and their key interactions on grain yield were estimated by the following model:

1_a/b_: yield ∼ L + N + K + promiscuity*P*I +country[.year]_b_*[P]_a_*I, random = ∼ [country.year/(I + P)]_a_ +location.year/(I + P) + trial/(I + P) + rep + mpf

• Model 1_a_ corrects for the application lime (L) nitrogen (N) and potassium (K) fertiliser. Random effects of country.year, location.year and trial were included as well as their interactions with P and I.
• Model 1_b_ included country/year as fixed effect and excluded the fixed country term.

To estimate trial-level yields and responses on the trials containing treatments with and without inoculation, we fitted the models:

2_a/b_. yield ∼ L + N + K + [promiscuity*P*I]_b_ + [promiscuity]_a_*trial*I, random = ∼[trial:P]_b_ + rep+ mpf

• Model 2_a_ was used for the subset of trials for which the interaction between inoculation and promiscuity could be estimated at the individual trial/field level. For this model, plots without P were removed from the data due to avoid bias due to imbalance in P application across trials.
• Model 2_b_ was applied to obtain field level estimates of inoculation effects for the larger subset of trials that included those where the effect of promiscuity could not be estimated per field.

Similarly, for the subset of country/years in which both promiscuous and specific varieties were planted we estimated country/year specific effects of promiscuity by fitting the model:

3. yield ∼ L + K + N + promiscuity*P*I + promiscuity*country.year*I, random = ∼ country.year:P + year.location/(I + P)+ trial/(I + P) + rep + mpf

• Model 3 served to obtain estimates of the average country/year-level inoculation effects for the two variety types.

To quantify the contribution of variation in inoculant response to yield variation we fitted the model:

4. yield ∼ L + N + K + promiscuity*P*I, random = ∼[year*country/(I + P) + year*location/(I + P)]+ trial/(I + P) + rep + mpf

• In model 4, variance components were estimated for interaction of inoculant with year, country, country/year, location/year and trial (field). Significance of individual components was evaluated with a likelihood ratio test. The distribution of predicted field/trial level responses was obtained by omitting the terms between square brackets and fixing the random effects as estimated in the full model.

Spatial analysis of yield and inoculation response for unique geographic coordinates was done by first fitting model 1 with replacement of the fixed model term country*P*I by coordinate*I. To improve model performance, geographic coordinates were aggregated at a minimum distance of 0.01° (approximately 1 km). Resulting means and variances were then used in a mixed model with country as a fixed term, a unit random term as a nugget effect and with the residual variance modelled with a Matérn spatial covariance function. Observations in the model were weighted by the inverse of the variance for each observation. Significance of spatial autocorrelation of the response variable was confirmed by Moran’s I test as implemented in the R package *spdep*.

A number of geographic data products were used as potential explanatory factors in the above mixed model: Annual mean temperature, maximum temperature of the warmest month, annual precipitation, precipitation of the wettest quarter (1 km resolution, www.worldclim.org, Hijmans et al., 2005) and predicted values for soil pH and percent sand at 15 cm (ISRIC 250 m soil property maps, www.soilgrids.org). A Wald test was used to test for significance.

## 3 Results

### 3.1 Inoculation response and patterns of variation

**Fig. 2:**
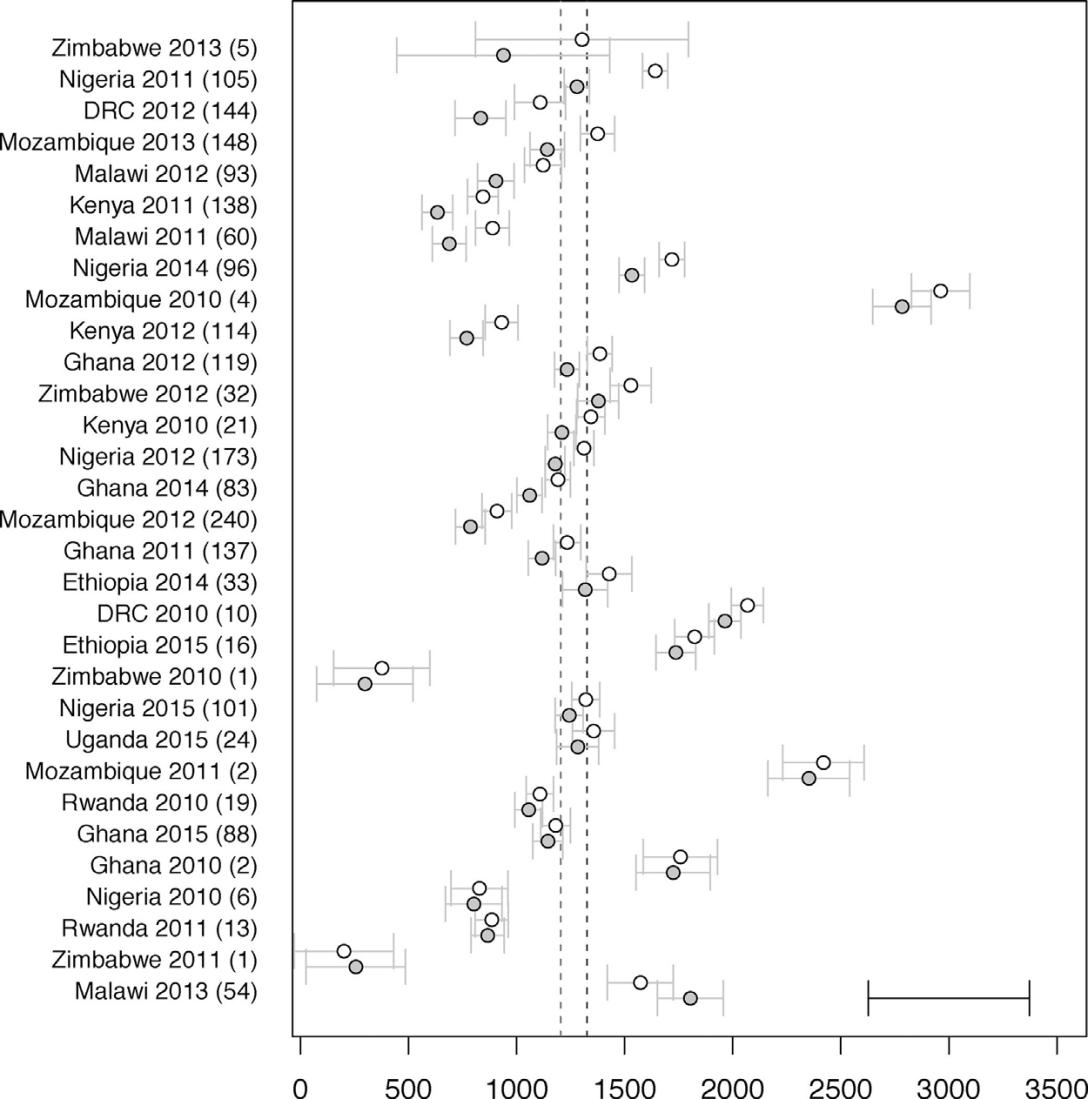
Average grain yields per country/year without inoculation (grey), with inoculation (white). Grey whiskers indicate standard errors of difference within country/year, black whiskers at the bottom right indicate the average least significant difference for between country/year comparisons. Entries are sorted vertically by magnitude of inoculation response. Numbers between parentheses indicate the number of trials for each country/year. Vertical grey/black dashed lines mark mean yields without/with inoculation.

The average response to inoculation over all trials was estimated at 115 kg/ha and was highly significant (model 1_a_, p < 0.0001). Estimated trial yields with and without inoculation averaged 1343 and 1227 kg/ha, respectively. Yield and inoculation response did not differ significantly between countries after correcting for years within country, but yields varied among year/country combinations (model 1_b_, p < 0.0001, Fig. 2). The strongest inoculation responses, in excess of 200 kg/ha, were observed in Kenya, Malawi and Nigeria in 2011, DRC and Malawi in 2012 and Mozambique and Zimbabwe in 2013. In contrast, responses of 25 kg/ha or less were observed for Nigeria in 2010, Zimbabwe and Rwanda in 2011 and Malawi in 2013. These differences were not significant after correcting for location/year (model 1_b_, p = 0.14), however, suggesting they may be attributed to local, rather than country-level differences. On average, addition of potassium, nitrogen and phosphorus significantly increased yields by 90, 80 and 129 kg/ha respectively (model 1_a_, p < 0.0025, 0.0001, 0.0001). Application of phosphorus had a significant interaction effect on inoculation response (p < 0.025) but the strength and direction of the interaction varied between countries (p < 0.0001).

**Fig. 3:**
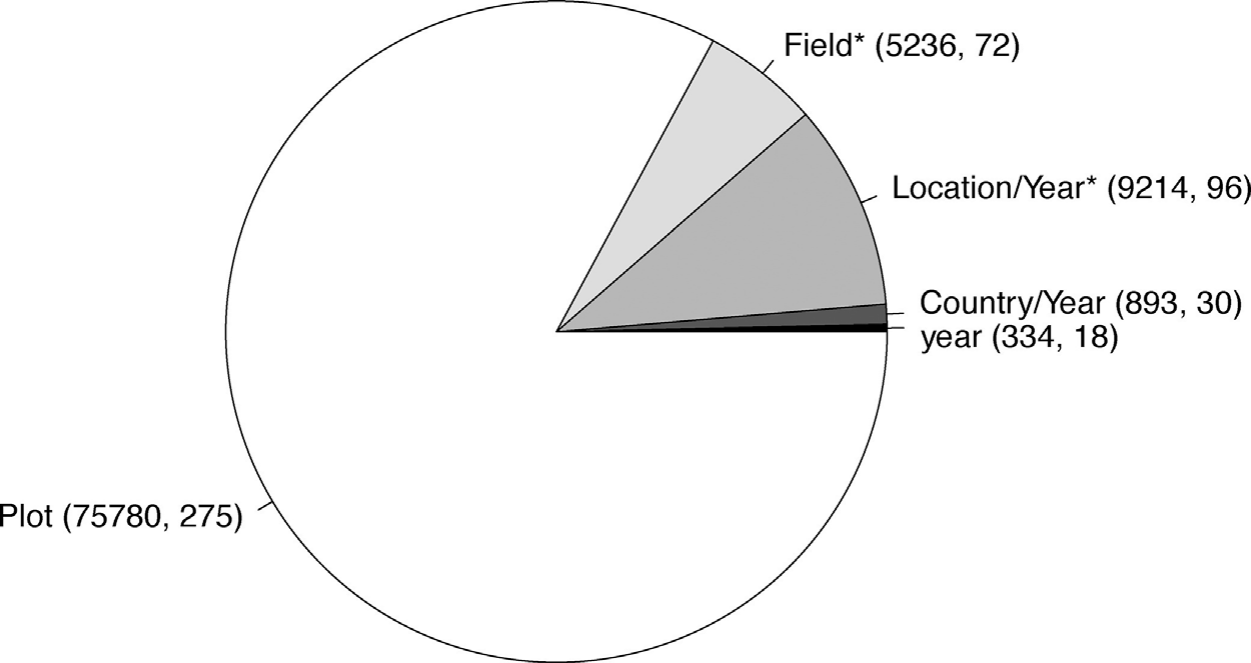
Breakdown of the contribution of different variance components related to inoculation response that contribute to yield variation. Only components larger than 0 are shown. Components significant at least the 5 percent level are indicated with an asterisk.

Overall, variation in inoculation response contributed considerably to yield variability, with significant variance components for location/year and fields (model 4, p < 0.0001), with variance components of 9223 and 5246 respectively. Most of the yield variation by far was due to unexplained plot-level variation, with residual variance estimated at 75,780 (Fig. 3). Inoculation response at the field level (estimated by model 2_b_) was significantly but weakly related to yield without inoculation (beta = −0.051, p < 0.00004, R^2^ = 0.015).

Variability of actual and observed response may affect famers’ willingness to adopt. Predicted variation at the trial/field level can be considered representative of variation in actual benefits obtained by individual farmers at a single field in a particular year, while residual variation at the experimental plot level is informative of the variability observed by farmers when evaluating a single unreplicated on-farm trial. We therefore looked at variation in predicted field- and observed plot level response in relation to two economic benchmarks, a benefit-cost ratio of two and a projected monetary profit of 100 USD per hectare. At the current price point of inoculants (Ronner et al., 2016) a benefit-cost ratio of 2 is reached at 25 kg/ha while 100 USD/ha profit is obtained for responses in excess of 180 kg/ha.

**Fig. 4:**
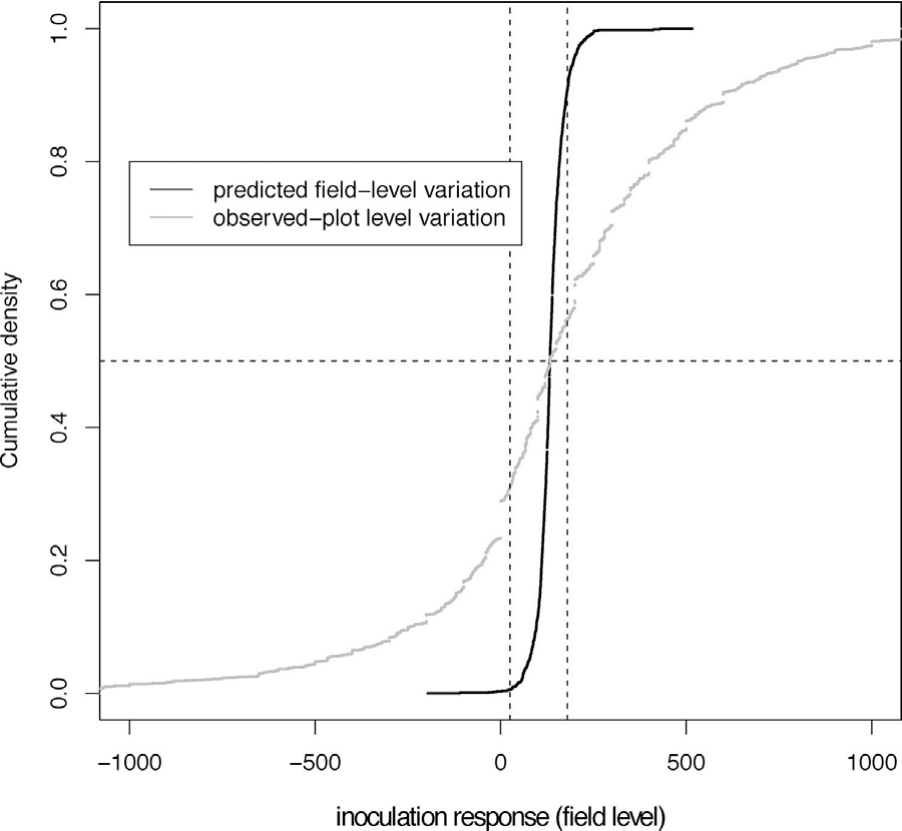
Cumulative distribution of predicted field-level responses (black) and observed responses at the experimental plot level (grey). Vertical dashed lines indicate the 25 kg/ha and 180 kg/ha points. The vertical dashed line marks the 50th percentile.

At the field level, less than three percent of farmers were predicted to have a yield response below 25 kg/ha while 20 percent were predicted to obtain a response in excess of 180 kg/ha (Fig. 4). Seventy five percent of farmers were predicted to have yield increases between 102 and 172 kg/ha, corresponding to a profit between 56 and 98 USD/ha.

Wide variability at the plot level meant that 44 percent of farmers with unreplicated trials were predicted to observe a response above 180 kg/ha while 39 percent would observe a response below 25 kg/ha (Fig. 4).

There was evidence for spatial autocorrelation of inoculation response and control yield, indicating that values were more similar at locations nearby (p < 0.0001). Autocorrelation decayed within distances of 10 to 20 km (Fig. S1), suggesting that spatial dependence is due to similarities at the district level and below. There was no significant relation between inoculation response or yield and any of the two soil properties (pH and percent sand) or climatic variables (annual mean temperature, maximum temperature of warmest month, annual precipitation).

### 3.2 The effect of promiscuity (all trials)

**Fig. 5:**
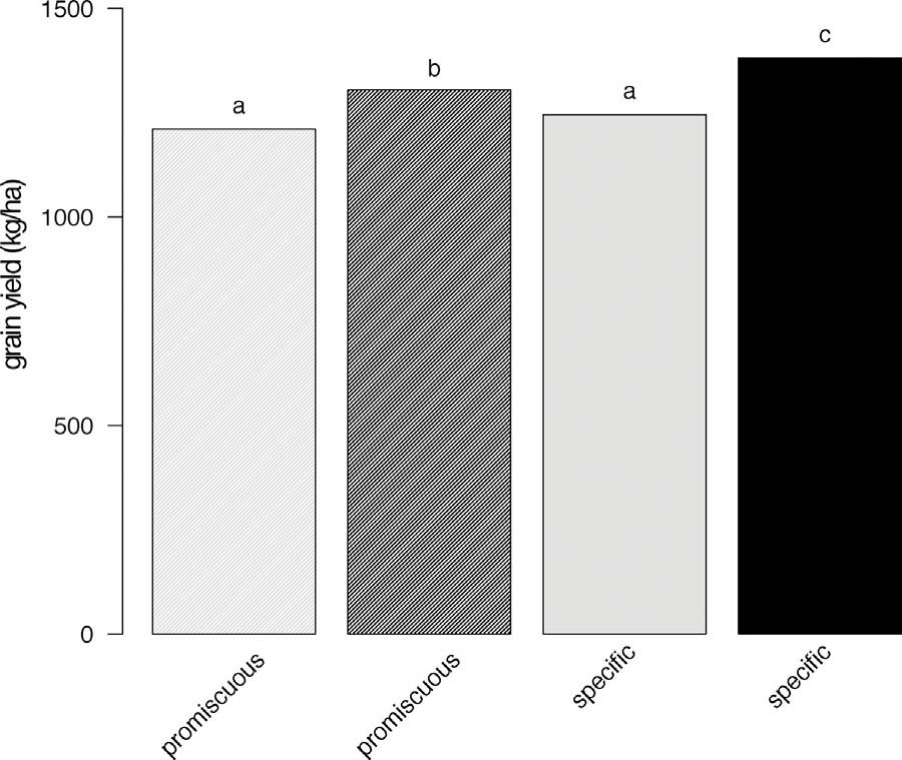
Average grain yields for promiscuous (shaded) and specific (solid) varieties without inoculation (grey), with inoculation (black). Whiskers indicate 2 times the average standard error of difference.

**Fig. 6:**
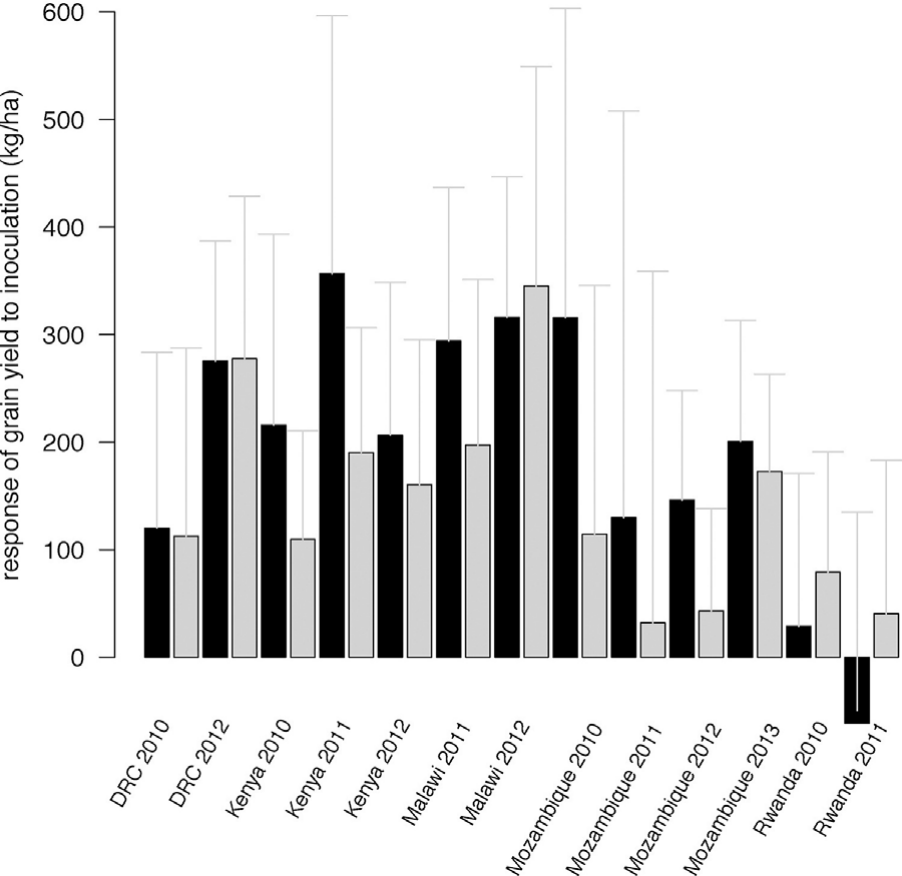
Inoculation responses (with P fertiliser) per country/year for promiscuous (black) and specific varieties (grey). Whiskers indicate standard errors.

Overall, there were 1638 trials with data on promiscuous varieties and 620 with data on specific varieties. Trials in Nigeria, Ghana and Uganda were planted exclusively to promiscuous varieties while in Ethiopia and Zimbabwe only specific varieties were planted. Across countries, yields without inoculation were similar for promiscuous and specific varieties (model 1_a_, p < 0.1), averaging 1211 and 1245 kg/ha respectively (Fig. 5). Yield after inoculation was significantly greater for both types of varieties (p < 0.0001) but response was significantly stronger for specific varieties (p < 0.001) which yielded an additional 42 kg/ha with inoculation, on top of the 94 kg/ha observed for promiscuous varieties. Consequently, specific varieties were estimated to yield 55 kg/ha more when averaged over inoculated and uninoculated conditions (p < 0.005). For the 421 trials where the two variety types could be compared at the country/year level, mean response was stronger for specific varieties in nine out of thirteen comparisons and yields of specific varieties were higher by 62 kg/ha without inoculation (model 3, p < 0.01), 113 kg/ha with inoculation (p < 0.0001) and 88 kg/ha on average (p < 0.0001; Fig. 6). For biomass, which was recorded in a subset of agronomy trials, a stronger inoculation response for specific varieties was also observed (p < 0.025), although biomass did not differ significantly between variety types, either on average (p < 0.8) or with or without inoculation (p < 0.06 and p  < 0.3 for uninoculated and inoculated respectively).

### 3.3 The effect of promiscuity (local effects)

**Fig. 7:**
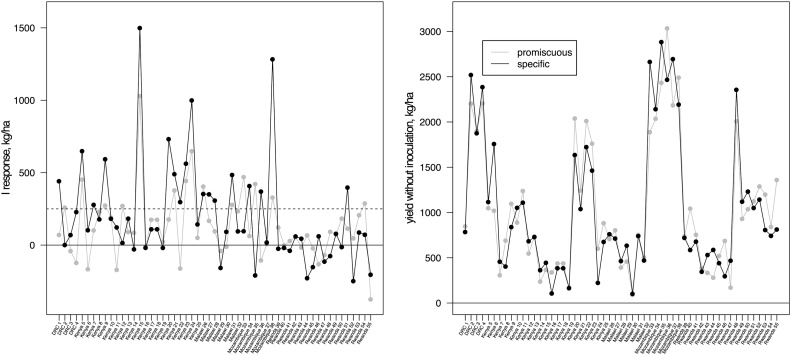
Mean inoculation responses (left panel) and yields (right panel) for individual trials containing two inoculation levels and both promiscuous (grey) and specific (black) varieties. P fertiliser was applied to all plots. The horizontal, dashed line marks a response of 250 kg/ha.

In a subset of 55 agronomy trials, promiscuous and specific varieties were grown in the same field with or without inoculation, providing information on site-specific performance of both variety types. On average, these trials confirmed the stronger inoculation response of specific varieties (model 2_a_, 202 vs 135 kg/ha, p < 0.02) and similar performance (1040 vs 1050 kg/ha, p < 0.7) without inoculation (Fig. 7). There was no overall yield benefit for any of the two variety types when averaged over inoculation levels (p < 0.8). Variation was large across sites, and specific varieties had greater response in only 30 trials (55%). Superior response of specific varieties was most evident in trials with estimated maximum responses above 250 kg/ha, for which specific varieties responsed more strongly in 17 out of 23 cases (74%) and mean response was larger by 175 kg/ha (456 vs 281 kg/ha, p < 0.001). Yields without inoculation remained similar (1311 vs 1346 kg/ha, p < 0.45) and average yields across the two inoculation levels were significantly greater for specific varieties (p < 0.001).

**Fig. 8:**
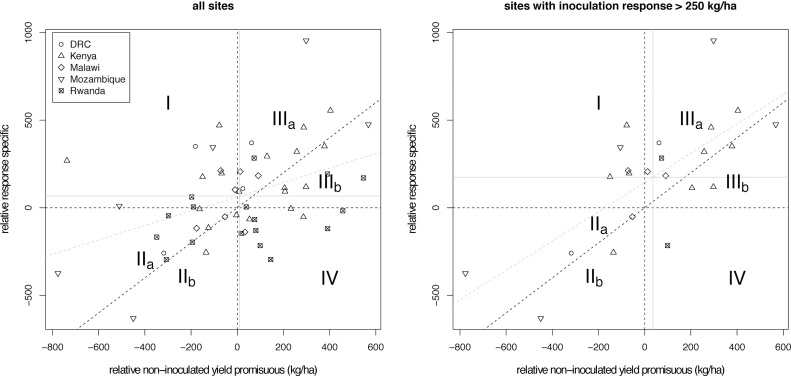
Scatterplot, based on the means from Fig. 7., showing the relative inoculation response of specific varieties (compared to the corresponding promiscuous variety), against the relative yield of promiscuous varieties without inoculation (compared to the corresponding specific variety). The vertical dashed line represents zero yield difference without inoculation, the dashed horizontal line marks zero difference in response, and the black dashed diagonal line marks the 1:1 line where a difference in yield is compensated by a difference in response of the alternative variety. Grey vertical and horizontal solid lines mark the means for relative yield and relative response respectively. The grey, dashed lines show the regression line of relative response against relative yield. Left panel: values for all samples. Right panel: values for subset of samples with an inoculation response above 250 kg/ha.

The above results can be represented more clearly by looking at the superiority of the two variety types in the different trials both with and without inoculation. This can be done by plotting the absolute difference in yield without inoculation against the absolute difference in response to inoculation (Fig. 8). Taking the 55 per-trial estimates as accurate means for the different sites, this shows directly which type of variety would work best at each site depending on the application of inoculant. Without inoculation, superiority is determined by uninoculated yield only. Sites where promiscuous varieties are superior are therefore located on the right side of the vertical axis (sections IIIa, IIIb and IV) whereas sites favouring specific varieties are on the left (sections I, IIa, and IIb). With inoculation, a variety is superior if its inoculation response is large enough to compensate its relative disadvantage in terms of uninoculated yield. Sites where specific varieties perform best are thus located above the 1:1 diagonal (sections I, IIa and IIIa), whereas sites where promiscuous varieties perform best are located below it (quadrants IIb, IIIb and IV).

Without inoculation, promiscuous varieties were superior in 31 out of 55 sites (56%), while with inoculation they performed best in 24 sites (Fig. 8, left panel). Reasons for superior performance of specific varieties with inoculation were superior response (11 sites, section IIIa), superior yield without inoculation (9 sites, section IIa) or both (11 sites, section I). Similarly, promiscuous varieties were favoured under inoculated conditions in sites where they showed high yields (8 sites, IIIb), strong response (4 sites, IIb), or both (12 sites, IV). Although the spread of sites across different superiority outcomes is rather even, there is a significant, positive correlation between the strength of response in specific varieties and yield superiority of promiscuous varieties without inoculation (beta = 0.41, R^2^ = 0.19, p < 0.001). In other words, specific varieties have the largest relative response in sites where they have the lowest relative yield without inoculation. This pattern is even clearer among the 23 sites with strong inoculation response (Fig. 8, right panel) where twelve out of thirteen sites (92%) with superior uninoculated yield of the promiscuous variety also show superior response of the specific variety and where conversely at five out of six sites with inferior uninoculated yield for promiscuous varieties specific varieties have inferior responses. Correlation is strong and highly significant with beta = 0.84, R^2^ = 0.51 and p < 0.0001. The observed correlation is expected if yield of specific varieties is limited by the lack of compatible, effective rhizobia in certain sites. Consistent with this interpretation, a subset of 7 out of the 23 trials with data on nodulation show higher nodulation scores for promiscuous varieties without inoculation (P < 0.0001) and similar scores when inoculant is applied (P < 0.26).

## 4 Discussion

Improving rhizobial nitrogen fixation in grain legumes is expected to offer tangible benefits to smallholder farmers in terms of increased production and enhanced residual soil fertility for subsequent crops (Giller and Cadisch, 1995). In soyabean, using productive varieties that can establish effective symbiosis and inoculating with elite rhizobium strains are possibly the most cost-effective ways of achieving such improvement. Our results confirm that on average, inoculation has a positive effect on grain yield, both for specific and promiscuous varieties, although average responses were moderate compared to earlier estimates from SSA (Pulver et al., 1982, Ronner et al., 2016) and the benefit of inoculation was minimal in some specific cases.

The extent and pattern of variation in response is relevant for the potential demand for inoculant in smallholder agriculture, since adoption will depend on farmers’ experience with the product. As in previous studies (Ronner et al., 2016), we observe substantial and statistically significant variation in response, particularly due to differences between locations/years and trials/fields. Field-level variability translates directly into variation in direct benefits obtained by different farmers in a given season. We find that despite substantial variation, responses for only few farmers are predicted to fall beneath a benefit-cost ratio of two, although conversely, only a small proportion of farmers is expected to obtain a monetary benefit above 100 USD/ha. Whether a profit between 56 and 98 USD/ha, as predicted for 75% of farmers would be sufficient for wide-scale adoption of inoculant remains open for debate.

The substantial plot-level variation reported here is relevant for the variation in response that is expected to be observed by farmers in unreplicated demonstration trials. The fact that observed responses in 30 percent of comparisons were below 25 kg/ha suggests that evaluation by farmers should be done using multiple plots, either at the same site or in different farmers’ fields, or potentially on larger areas, which may also reduce variability. Ideally, inoculant use would be targeted based on predictions of their effectiveness at different locations. We show that in spite of the existence of significant geographic variation, we cannot make predictions based on available biophysical data. In fact, the lack of correlation between inoculation response and yield, suggests that even yield constraints may not be strong predictors of the effect of inoculation. This result confirms earlier reports of limited predictability of inoculation response (Ronner et al., 2016) and suggests that other factors such as history of soyabean cultivation may play a role (de Bruin et al., 2010), or that we simply do not have sufficiently accurate data on agronomic, environmental and edaphic conditions to contribute to the prediction of response given current levels of variability. A further confounding factor in our analysis is that the commercial inoculants available varied across the countries, both in terms of inoculant strains and their formulations. Experiments addressing this issue are currently ongoing.

Our study presents the first comparative analysis of the performance of promiscuous and specific varieties across SSA. We find that on average, promiscuous varieties do not yield more in the absence of inoculation and that specific varieties respond more strongly to inoculation. This result is somewhat unexpected, given that the premise behind breeding for promiscuity is improved performance under non-inoculated conditions. Recent work in Mozambique (Gyogluu et al., 2016) yielded similar results locally but here we observe the same pattern in different countries and across different scales. Our breakdown of site-specific responses provides insight into the differential performance of the two types of varieties at different sites. Over all trials, there is an even distribution of superiority in terms of uninoculated yield and response, suggesting that either type could be superior depending on location and application of inoculant. This is partly due to the occurrence of sites with low inoculation responses, which may reflect the presence sufficient rhizobia or soil nitrogen, or poor quality and ineffective inoculant. The latter would be a more likely explanation wherever low responses and superior uninoculated yields of promiscuous varieties co-occur. Where inoculation has a strong effect, there is a clear tendency for increased response of specific varieties to coincide with superior yields of promiscuous varieties. This, combined with the higher nodulation scores in promiscuous varieties, points to the abundance of compatible and effective rhizobia as a yield limiting factor at those sites and as a determinant of the magnitude of inoculation response. The fact that specific varieties generally respond more strongly, suggests they nodulate preferentially with the inoculant strain, whereas promiscuous types also recruit more abundant, but less effective local strains, even when inoculated. Molecular ecological work on bacterial populations in both types of varieties could shed light on this matter.

It is important to note here that an older study comparing the two types of varieties (Pulver et al., 1982), while reporting poor nodulation and poor yields in uninoculated, specific varieties, included a specific variety with good yield and response. Undoubtedly the relative performance of promiscuous types depends on the varieties being compared. Among modern varieties, specific types may therefore perform better than expected based on older variety comparisons.

Our results suggest that specific types should be preferred if suitable varieties exist, regardless of the availability of inoculant. On the other hand, the absolute gain in terms of extra response is relatively limited, at an estimated 25 USD/ha, and is likely to be overshadowed by site-specific differences such as those reported here. Also, response to inoculation and grain yield are not the only attributes relevant to variety selection. Promiscuous and specific soyabean have had different breeding histories, with an emphasis on the development of dual purpose varieties in the case of promiscuous varieties (Tefera, 2011). The two variety types are therefore likely to differ in a range of characteristics including biomass (Mpepereki et al., 2000), resistance to specific diseases (Tefera, 2011), grain size, and oil contents (Njoroge et al., 2015). It will therefore be the appropriate targeting of varieties that is likely to provide the highest gains, the challenge remaining to predict the suitability of technologies for smallholder farmers in the face of overwhelming variability.
