# Supplementary material for: Soyabean response to rhizobium inoculation across sub-Saharan Africa: Patterns of variation and the role of promiscuity
Source: Agric Ecosyst Environ. 2018 Jul 1;261:211–8. doi: 10.1016/j.agee.2017.08.016 (PMC5946711; doi:10.1016/j.agee.2017.08.016)
Supplement: Supplementary file 1 [file mmc1.doc]

Table S1. Overview of agronomy trials

| **country** | **type(s) of p-fertilizer** | **Other inputs** | **inoculant type/brand** | **inoculant strain** | **carrier material** | **trial design** | **trial type** |
| --- | --- | --- | --- | --- | --- | --- | --- |
| DRC | TSP, DAP |  | BIOFIX | USDA 110 | Sterile filter mud | Split plot | input |
| Ghana | TSP, SSP, YARA Legume (NPK 0:18:12 plus Mg, Zn, Mo and Bo) | Fertsol | LEGUMEFIX | 532c | Irradiated peat | Split plot | input |
| Kenya | TSP, DAP, MRP, SYMPAL (NPK 0:23:15 plus 10% Ca, 4% S, 1% Mg and 0.1% Zn) | Lime | BIOFIX | USDA 110 | Sterile filter mud | Split plot | input |
| Malawi | TSP, Compound D (NPK 10-20-10 + 6.5 S) |  | BIOFIX | USDA 111 | Sterile filter mud | Split plot | input |
| Mozambique | SSP |  | Soygro Soycap | WB74 | Sterile clay | Split plot | input |
| Nigeria | SSP, RP | Agrolyser | LEGUMEFIX | 532c | Irradiated peat | Split plot | input |
| Rwanda | TSP, DAP, MRP, SYMPAL (NPK 0:23:15 plus 10% Ca, 4% S, 1% Mg and 0.1% Zn) |  | BIOFIX | USDA 110 | Sterile filter mud | Split plot | input |
| Uganda | TSP |  | MAKBIOFIXER | USDA 110 | Sterile peat | Split plot | input |
| Zimbabwe | TSP. Compound D (NPK 10-20-10 + 6.5 S) |  | SPRL_product (no name) | MAR 1391 | Sterile bargasse | Split plot | input |
| DRC | TSP | MoP (KCl) | BIOFIX | USDA 110 | Sterile filter mud | Split plot | variety |
| Ghana | TSP | MoP (KCl) | LEGUMEFIX | 532c | Irradiated peat | Split plot | variety |
| Kenya | TSP | MoP (KCl) | BIOFIX | USDA 110 | Sterile filter mud | Split plot | variety |
| Malawi | TSP | MoP (KCl) | BIOFIX | USDA 110 | Sterile filter mud | Split plot | variety |
| Mozambique | SSP | ) | Soygro Soycap | WB74 | Sterile clay | Split plot | variety |
| Nigeria | SSP | MoP (KCl) | LEGUMEFIX | 532c | Irradiated peat | Split plot | variety |
| Rwanda | TSP | MoP (KCl) | BIOFIX | USDA 110 | Sterile filter mud | Split plot | variety |
| Uganda | TSP | MoP (KCl) | Bio-N-Fix | - | Sterile peat | Split plot | variety |
| Zimbabwe | TSP | MoP (KCl) | SPRL_product (no name) |  | Sterile bargasse | Split plot | variety |
| DRC | TSP | MoP (KCl) | BIOFIX | USDA 110 | Sterile filter mud | Split plot | Need to inoculate |
| Ghana | TSP |  | LEGUMEFIX | 532c | Irradiated peat | Split plot | Need to inoculate |
| Kenya | TSP |  | BIOFIX | USDA 110 | Sterile filter mud | Split plot | Need to inoculate |
| Malawi | TSP | MoP (KCl) | BIOFIX | USDA 110 | Sterile filter mud |  | Need to inoculate |
| Mozambique |  |  |  |  |  |  | Need to inoculate |
| Nigeria | SSP, RP |  | LEGUMEFIX | 532c | Irradiated peat | Split plot | Need to inoculate |
| Rwanda | TSP |  | BIOFIX | USDA 110 | Sterile filter mud | Split plot | Need to inoculate |
| Zimbabwe |  |  | SPRL_product | MAR 1391 | Sterile bargasse | Split plot | Need to inoculate |
| Uganda | TSP |  | LEGUMEFIX, MakBiofixer | 532c, USD 110M | Irradiated peat, sterile peat | RCBD | Inoculation, input (try-outs) |
| Ethiopia | DAP |  | MBI inoculant | TAL379 | Lignite | RCBD | Inoculation, input (try-outs) |

Table S2. Overview of varieties used

| **Variety** | **Promiscuity** | **maturity** | **Countries** | **Line Name** | **released** | **origin** | **Pedigree** |
| --- | --- | --- | --- | --- | --- | --- | --- |
| 449/16 | No | Medium | DRC |  |  |  |  |
| belesa-95 | No | Medium | Ethiopia |  | 2003 | AARC/SARI | PR-149 |
| clark 63k | No | Late | Ethiopia |  | 1982 | AARC/SARI |  |
| dhidhessa | No | Late | Ethiopia |  | 2008 | BARC/OARI | PR-149-81-EP-7-2 |
| EAIi3600 (, Sb97) | No | Medium | Kenya | - | 1996 |  |  |
| imperial | No | early | DRC |  | 1977 |  |  |
| makwacha | No | Early | Malawi |  | 2008 | Malawi |  |
| Nasoko | No | Early | Malawi | 427/5/7 | 1993 | Malawi |  |
| PANn1867 | No | medium | Malawi |  |  |  |  |
| pk6 | No | Early | DRC | Peka 6 | 2004 | ISVEX (INDIA) |  |
| SC Safari | No | SC Safari | Mozambique, Zimbabwe |  | 2001 | Seed Co, Zimbabwe |  |
| SC Santa | No | SC Santa | Mozambique | Santa | 1981 | Brazil |  |
| Sc saga | No | medium | Malawi, Zimbabwe | Saga | 2008 | Seed Co, Zimbabwe |  |
| Sc squire | No | medium | Zimbabwe, Kenya | Squire | 2008 | Seed Co, Zimbabwe |  |
| SC Serenade | No | Medium | Mozambique, Zimbabwe | Serenade | 2008 | Seed Co, Zimbabwe |  |
| SC Solitaire | No | medium | Malawi | Solitaire | 2003 | Seed Co, Zimbabwe |  |
| SC Soprano | No | medium | Malawi | Soprano | 2003 | Seed Co, Zimbabwe |  |
| soprosoy | No | Early | Rwanda | Soprosoy | 2004 | ISVEX (INDIA) |  |
| SC Storm | No | Early | Mozambique | Storm | 2001 | Zimbabwe |  |
| Yezumutima | No | medium | Rwanda |  |  |  |  |
| Anidaso | Yes | Early | ghana | TGx 813-6D | 1992 | IITA | TGM 1197 x TGM 618 |
| jenguma | Yes | late | ghana | TGx 1448-2E | 2003 | SARI, IITA | TGX 824-18D x TGX 814-27D |
| maksoy2n | Yes | Medium | Uganda |  | 2008 | MAK |  |
| maksoy3n | Yes | Medium | Uganda |  | 2010 | MAK |  |
| maksoy4n | Yes | Medium | Uganda |  |  | MAK |  |
| maksoy5n | Yes | Medium | Uganda |  |  | MAK |  |
| ocepara | Yes |  | Malawi | Ocepara-4 | 1993 | Argentina |  |
| Sb24 | Yes | Medium | DRC, Rwanda |  |  |  |  |
| Sb25 | Yes | Medium | Kenya | Namsoy 4M |  |  |  |
| Sb3 | Yes | Medium | Kenya | TGx 1835-10E | Informal | IITA | TGx 1213-1D x TGx 1445-3E |
| Sb8 | Yes | Medium | Kenya |  |  |  |  |
| sungpungu | Yes |  | Ghana |  |  |  |  |
| TGx 1448-2E | Yes | Late | ghana, nigeria | TGx 1448-2E | 1994 | IITA | TGx 824-18D x TGx 814-27D |
| tgx-1485-1d | Yes | Early | Mozambique | TGx 1485-1D | 2011 | IITA | TGx 316-024Dx TGx 813-11D |
| TGx 1740-2F, sb19, Tikolore or Wamini | Yes | medium | DRC, Kenya, Malawi, Mozambique, Zimbabwe, Rwanda | TGX 1740-2F | 2007  2011 in Malawi and Mozambique | IITA | TGx 539-5E x SIBLEY |
| TGx 1835-10E | Yes | early | ghana, Malawi, nigeria | TGx 1835-10E | Nigeria | IITA | TGx 1213-1D x TGx 1446-1E |
| TGx 1904-6F | Yes | late | Mozambique, nigeria | TGx 1904-6F | 2009  2011 (Mozambique) | IITA & NCRI | TGx 1802-2F X TGx 1834-5E |
| TGx 1908-8F | Yes | late | Mozambique | TGx 1908-8F | 2011 | IITA | TGx 1829-1E x TGx 1834-5E |
| TGx 1935-3F | Yes | late | nigeria |  |  |  |  |
| TGx 1937-1F | Yes | late | Mozambique | TGx 1937-1F | 2011 | IITA | TGx 1805-31F x TGx 1834-5E |
| tgx-1945-1f | Yes | late | nigeria |  |  |  |  |
| tgx-1951-3f | Yes | late | nigeria |  |  |  |  |
| tgx-1963-3f | yes |  |  |  |  |  |  |
| tgx-1987-10f | Yes | late | Malawi | TGx 1987-10F | 2010 | IITA | TGx 1805-31F x UG5 |
| tgx-1987-11e | Yes | late | Zimbabwe |  |  |  |  |
| tgx-1987-11f | Yes | late | Malawi, Rwanda |  |  |  |  |
| tgx-1987-18f | Yes | medium |  |  |  |  |  |
| tgx-1987-20f | Yes | late | Rwanda |  |  |  |  |
| tgx-1987-28f | Yes | late | Rwanda |  |  |  |  |
| tgx-1987-38f | yes |  |  |  |  |  |  |
| tgx-1987-57f | yes |  |  |  |  |  |  |
| tgx-1987-62f | Yes | late | Malawi, Rwanda | TGx 1987-62F | 2010 | IITA | TGx 1805-31F x UG5 |
| tgx-1987-64 | Yes | late | Rwanda |  |  |  |  |

Figure S1. Variogram showing the spatial autocorrelation of the predicted inoculant response . The solid line indicates the modelled variogram based on the estimated covariance parameters.
